# A rare cause of urinary retention in A child: A case report

**DOI:** 10.1016/j.eucr.2023.102328

**Published:** 2023-01-17

**Authors:** Maher Al-Hajjaj, Ahmed Tawosh, Ahmed Osman Adam Bary, Abdilya Riyadh Alabdaly, Razan Osama Altayep

**Affiliations:** a Department of Urology, Aleppo University Hospital University of Aleppo, Aleppo, Syria; b University of Harran, Turkey; c University of Kassala, Kassala, Sudan; d Jordanian University, Amman, Jordan; e Al Yarmouk Faculty of Medical Science, Khartoum, Sudan

## Abstract

A Ten-year-old child presented to our emergency department with acute urinary retention. Three months ago, he was diagnosed with right renal hydronephrosis with renal calculus. Firstly, right nephrostomy was inserted. Next, he had been treated with ESWL for right renal stone in another center. After unsuccessful attempts for urethral catheterization, he was brought to the operating room for meatotomy and calculus extraction. An impacted urethral stone was detected at this point. He had a successful extraction of the stone. A six-month follow-up period has passed with no recurrence. This shows that although they are uncommon, urethral stones do happen.

## 1 Introduction

Urethral stones are very rare localization of urinary stones, and they are in most cases located at the posterior urethra, most often secondary to the migration of bladder stones or upper urinary tract stones, and rarely formed primarily in the urethra, they are relatively more frequent in childhood and rare in females, its clinical manifestations vary widely, ranging from simple and progressive dysuria to acute retention of urine or even more serious complications^1^^,^^2^

We had a rare case of acute urinary retention in a child due to urethral stone.

Successful treatment with meatotomy was achieved.

## 2 Case presentation

**Fig. 1:**
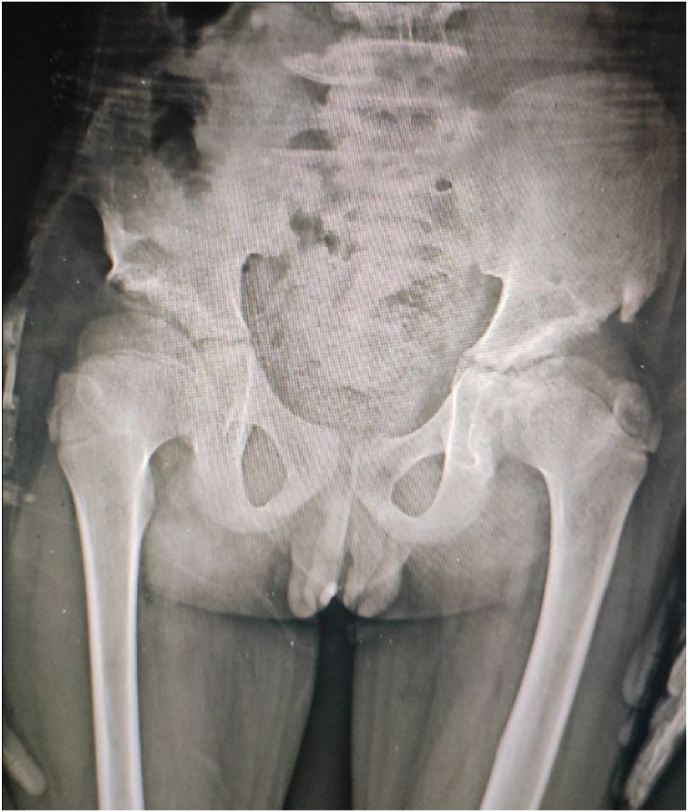
KUB image shows urethral stone.

A ten-year-old male came to our department of emergency for the first time for urinary retention. Past medical history revealed a right renal stone discovered after flank pain two months ago. He underwent a right nephrostomy and ESWL for the stone in another center. His only drug is Ibuprofen. Physical examination showed lower abdominal tenderness and fullness. His Laboratory findings demonstrated leukocytosis (12.5, 000/μl) with normal renal function and a normal hemoglobin level. Ultrasonography of the abdomen and pelvis showed normal kidneys with a full bladder in urine. A plain x-ray of the abdomen and pelvis demonstrates opacity in the anterior urethra suggesting a urethral stone (Fig. 1). Inserting a urinary catheter or pushing the stone back failed to relieve the retention.

After taking the parent's consent, we decided to perform meatotomy.

Under general anesthesia, we performed a meatotomy. We extracted the stone. About 350 ml of urine was drained in a urine bag. Closing the incision with 5/0 vicrel. The post-surgery course was uneventful.

The child was discharged the day after the surgery. He had no complaints during the 3 months of follow-up.

## 3 Discussion

The incidence of urethral stones was reported 7 per 100.000, with the male predominance. The male to female ratio is 13:1.^3^

As a rare condition, urethral calculi can be classified into two groups: native or migrant.

Native urethral calculi are secondary to abnormalities that predispose to urinary stasis and infection.

Migrant urethral calculi, is more common condition, a calculus formed in the kidney or bladder moves to and blocks the urethra. Migrant calculi can cause pain, feeling of pelvic pressure, acute urinary retention and irritative symptoms.^4^

The diagnosis is most often easy by interrogation which seeks the history of urinary stones disease or emission of calculus, penile pain, and by the clinical examination which made it possible to palpate a mobile hard urethral mass when it is not impacted and located at the anterior urethra. In other cases, the diagnosis can be confirmed by a simple radiography centered on the external genitalia or a penile ultrasound in the case of radiolucent stones which are not rare.^5^

We presented a rare cause of urinary retention in a child with a history of renal calculi and ESWL. The stone moved from the upper urinary tract to the urethra. It caused the boy to have a difficult and painful urination. The confirmed diagnosis was by the plain x-ray. Meatotomy and stone extraction was the definitive treatment.

## 4 Conclusion

Urethral stone is considered a very rare cause of urinary retention. Diagnosis of such cases is easy by medical history and physical examination. A plain x-ray may be needed to confirm the diagnosis. Extraction of the calculus by meatotomy was the treatment.

## Funding

This research did not receive any specific grant from funding agencies in the public, commercial, or not-for-profit sectors.

## Declaration of competing interest

None.
